# *Campylobacter jejuni* in Hospitalized Patients with Diarrhea, Kolkata, India

**DOI:** 10.3201/eid1907.121278

**Published:** 2013-07

**Authors:** Piyali Mukherjee, T. Ramamurthy, Mihir K. Bhattacharya, K. Rajendran, Asish K. Mukhopadhyay

**Affiliations:** Author affiliation: National Institute of Cholera and Enteric Diseases, Kolkata, India

**Keywords:** Diarrhea, Campylobacter jejuni, antimicrobial resistance, India, bacteria

**To the Editor:** *Campylobacter* spp. infection is the leading cause of bacterial enteritis worldwide. The epidemiology of *Campylobacter* infection in developing countries differs substantially from that in industrialized countries. In many studies from the United States and other industrialized countries, *Campylobacter* spp. are among the most common bacterial causes of diarrhea (*1*), with an incidence of ≈10% in persons with diarrhea. Reports from developing countries also suggest that *C. jejuni* and *C. coli* have been isolated mostly from patients with diarrheal illness (*2*). We investigated for the prevalence of *Campylobacter* infection in patients hospitalized with diarrhea at the Infectious Disease Hospital in Kolkata, India, and their resistance patterns to different antimicrobial drugs.

During January 2008–December 2010, we screened 3,186 fecal samples on brain–heart infusion agar with 5% defibrinated sheep blood and antimicrobial drugs (bacitracin, cycloheximide, colistin sulfate, cephazoline sodium, novobiocin) and incubated under microaerophillic environment (5% O_2_, 10% CO_2_, and 85% N_2_) at 37°C for 48 h. Each isolate was tested by Gram staining, cytochrome oxidase, and hippurate hydrolysis for presumptive identification and species-specific PCR (*3*) to identify 5 species from *Campylobacter* genus. The overall isolation rate of *Campylobacter* spp. was ≈7% (222/3,186). Sole infection with *Campylobacter* spp. accounted for only 40% of cases; others were mixed infection. *C. jejuni* was the predominant species (78%), with *C. coli*, *C. fetus*, *C. lari*, and *C. upsaliensis* isolated less frequently. *Campylobacter* infection prevailed throughout the year, with no seasonality. The *C. jejuni* isolation rate was significantly higher (10.0%; p<0.001) for children <5 years of age who had diarrhea than for persons in other age groups (3.7%). Although we used the culture method, which is the standard for screening fecal samples, some molecular methods, such as PCR and real-time PCR, are now used for screening *Campylobacter* spp. from fecal samples on the Indian subcontinent (*4*,*5*). The results from molecular methods are showing more infection with *Campylobacter* spp. and high mixed infection cases and suggest the usefulness of molecular methods in combination with cultures.

Macrolides and fluoroquinolones generally are the first- and second-line choices, respectively, of antimicrobial drugs for treating *Campylobacter* enteritis. Since the late 1980s, resistance to these drugs has complicated treatment. In India, resistance of *Campylobacter* spp. to several antimicrobial drugs has been reported since the early 1990s (*6*). Fluoroquinolone resistance was not reported in 1994 but reached 79% during 2001–2006 (*2*). Likewise, ciprofloxacin resistance in *Campylobacter* spp. increased markedly in Dhaka, Bangladesh, during 2005–2008 (*7*) and in Karachi, Pakistan, during 1992–2002 (*8*). We tested 142 *C. jejuni* isolates for antimicrobial susceptibility by disk diffusion method on Muller-Hinton agar with 5% sheep blood and incubated them at 37°C in microaerophillic environment for 48 h before obtaining results. All tested strains were resistant to trimethoprim–sulfamethoxazole, and most (97%) were resistant to quinolone (nalidixic acid) and fluoroquinolones (norfloxacin, ciprofloxacin, and ofloxacin) (Figure). Approximately 26.1% and 17.6% of the isolates were resistant to ampicillin and tetracycline, respectively. Susceptibility to erythromycin, azithromycin, gentamicin, furazolidone, and chloramphenicol was very high (>97%) in most isolates. Multidrug resistance was frequent among many of the isolates: ampicillin, nalidixic acid, norfloxacin, ciprofloxacin, ofloxacin, and trimethoprim–sulfamethoxazole (19%); tetracycline, nalidixic acid, norfloxacin, ciprofloxacin, ofloxacin, and trimethoprim–sulfamethoxazole (10.2%); and tetracycline, ampicillin, nalidixic acid, norfloxacin, ciprofloxacin, ofloxacin, and trimethoprim–sulfamethoxazole (6.8%). These results indicate that macrolides may be useful for treating campylobacteriosis in this region.

**Figure:**
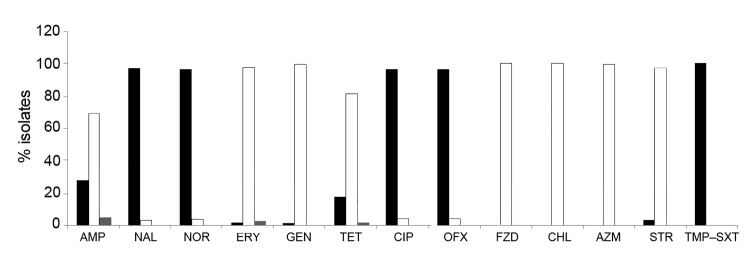
Antimicrobial drug susceptibility profile of 142 *Campylobacter jejuni* isolates, Kolkata, India, 2008–2010. Black bars, resistant; gray bars, intermediate resistance; white bars, susceptible. AMP, ampicillin; NAL, nalidixic acid; NOR, norfloxacin; ERY, erythromycin; GEN, gentamicin; TET, tetracycline; CIP, ciprofloxacin; OFX, ofloxacin; FZD, furazolidone; CHL, chloramphenicol; AZM, azithromycin; STR, streptomycin; TMP–SXT, trimethoprim–sulfamethoxazole.

The resistance patterns are influenced by various factors, possibly including pressure exerted by use of antimicrobial drugs. Various reports have stated that introduction of fluoroquinolones for use in veterinary practice has been associated with a dramatic rise in *Campylobacter* strains showing resistance to these drugs (*9*). Increasing antimicrobial drug resistance limits the number of therapeutic options, which makes empirical treatment more difficult. Therefore, constant monitoring of *Campylobacter* susceptibility to antimicrobial agents is essential. We could not detect any allele of plasmid-mediated quinolone resistance genes (*qnr*) among *C. jejuni* isolates and the different class of mobile genetic elements that generally carry the antimicrobial resistance gene cassettes. However, we found that most of the *C. jejuni* isolates had a mutation in the quinolone-resistance determining region of *gyrA* (Thr-86 to Ile), which led the isolates to become resistant for quinolone and fluoroquinolones.

Recent microbiome analysis of the gut of a malnourished child residing in an urban slum in Kolkata showed 35 times more *Campylobacter* bacteria than in healthy child in the same setting (*10*). This finding suggests that intestinal inflammation may directly influence malabsorption of nutrients. Hence, it is essential to examine the effect of *Campylobacter* infection in the developing world in the context of many recent developments in the human gut microbiome.
